# Hierarchical Hydrogels with Ordered Micro-Nano Structures for Cancer-on-a-Chip Construction

**DOI:** 10.34133/2021/9845679

**Published:** 2021-12-26

**Authors:** Luyao Zhu, Changmin Shao, Hanxu Chen, Zhuoyue Chen, Yuanjin Zhao

**Affiliations:** ^1^Department of Clinical Laboratory, Institute of Translational Medicine, The Affiliated Drum Tower Hospital of Nanjing University Medical School, 210008 Nanjing, China; ^2^State Key Laboratory of Bioelectronics, School of Biological Science and Medical Engineering, Southeast University, Nanjing 210096, China; ^3^Oujiang Laboratory (Zhejiang Lab for Regenerative Medicine, Vision and Brain Health), Wenzhou, Zhejiang 325001, China; ^4^Chemistry and Biomedicine Innovation Center, Nanjing University, Nanjing 210023, China

## Abstract

In the drug therapy of tumor, efficient and stable drug screening platforms are required since the drug efficacy varies individually. Here, inspired by the microstructures of hepatic lobules, in which hepatocytes obtain nutrients from both capillary vessel and the central vein, we present a novel hierarchical hydrogel system with ordered micro-nano structure for liver cancer-on-a-chip construction and drug screening. The hierarchical hydrogel system was fabricated by using pregel to fill and replicate self-assembled colloidal crystal arrays and microcolumn array template. Due to the synergistic effect of its interconnected micro-nano structures, the resultant system could not only precisely control the size of cell spheroids but also realize adequate nutrient supply of cell spheroids. We have demonstrated that by integrating the hierarchical hydrogel system into a multichannel concentration gradients microfluidic chip, a functional liver cancer-on-a-chip could be constructed for high-throughput drug screening with good repeatability and high accuracy. These results indicated that the hierarchical hydrogel system and its derived liver cancer-on-a-chip are ideal platforms for drug screening and have great application potential in the field of personalized medicine.

## 1. Introduction

Liver cancer has been one of the most common and refractory diseases over the world due to its poor prognosis and high mortality. In clinical practice, numerous therapeutic methods such as surgical resection, drug therapy, chemotherapy, and radiotherapy have been adopted [1–3]. Among them, drug therapy is the main approach due to its simplicity and convenience [4–6]. However, the efficacy of these drugs varies individually in the patients with advanced stage of liver cancer because of the tumor heterogeneity [7–9]. Hence, a stable and efficient preclinical drug screening platform is urgently required. Recently, three-dimensional (3D) cell culture based on hydrogel system has gained great interest as a promising platform for anticancer drug screening [10–12]. Since the hydrogel system precisely mimics the natural extracellular matrix (ECM) environment, cells embedded in the hydrogel will produce endogenous ECM proteins and continue to aggregate to form cell spheroids [13–15]. However, the cell spheroids obtained by simple embedding method are usually with uneven size and poor monodispersity, which might affect the repeatability and accuracy of subsequent drug screening. In addition, due to the simple structure of these hydrogels, the cells in the core of some big spheroids are lacking of nutrition and prone to necrosis during the culture process, which would make the screening results unreliable. Therefore, the development of drug screening platform with more sophisticated structures and functions is still requisite.

**Figure 1:**
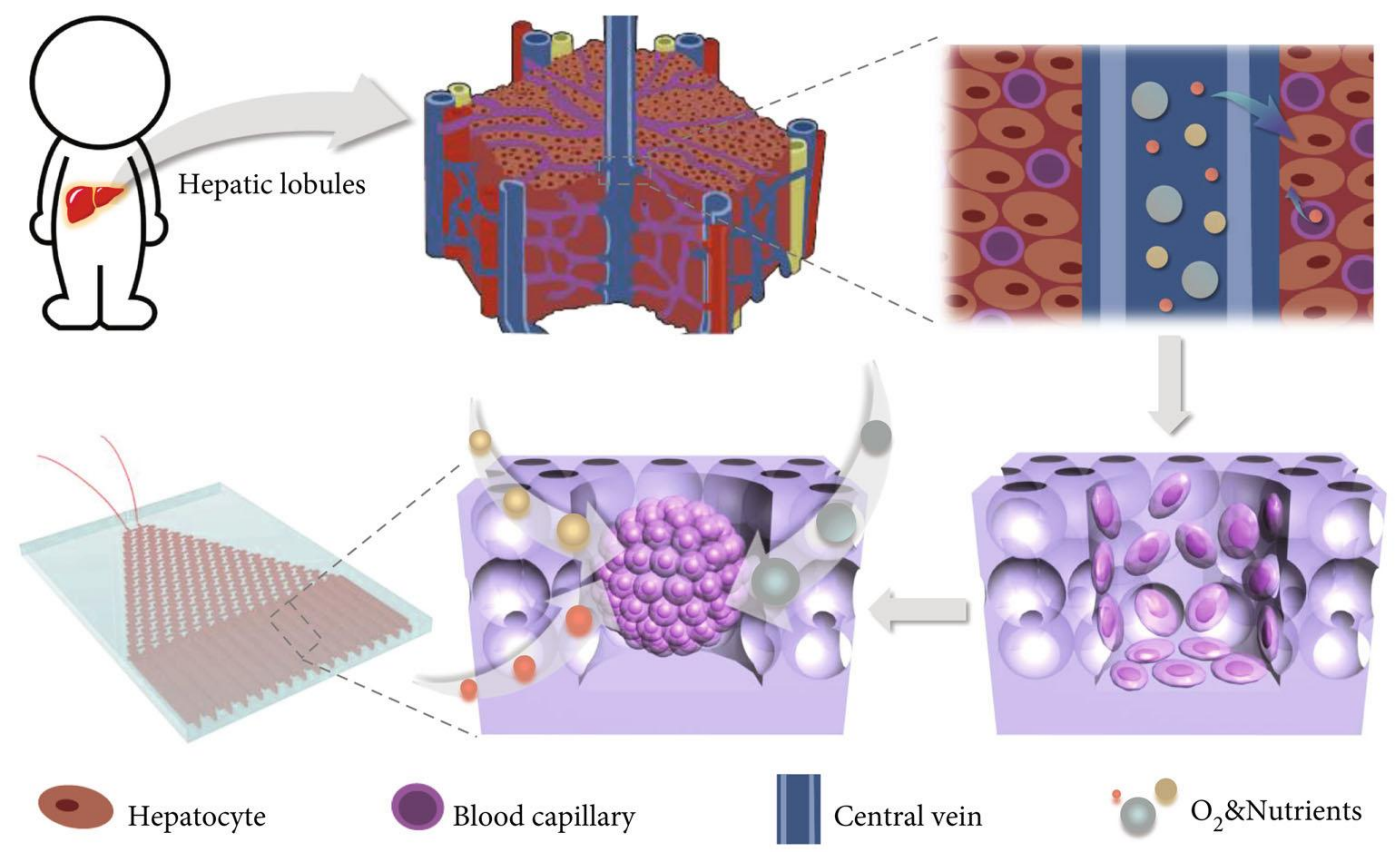
Mechanism of the hierarchical hydrogel with ordered micro-nano structure for liver cancer-on-a-chip construction.

Herein, inspired by the microstructures of hepatic lobule, we proposed a novel hierarchical hydrogel system with ordered micro-nano structure for liver cancer-on-a-chip construction and drug screening, as schemed in Figure 1. It is well known that the fundamental structural and functional unit of the liver is the polyhedral and prismatic hepatic lobule [16, 17]; in the center of that, a longitudinal central vein is distributed in which the hepatocytes arrange radially to form the hepatic plate. Between hepatic plates is hepatic sinusoid, which is a special capillary vessel that provides nutrients to hepatocytes together with the central vein [18, 19]. On the other hand, as a bionic system, the organ-on-a-chip can simulate the staple structure and function of different organs in vitro and even the relationship between multiple organs, so as to forecast the human body's feedback on drugs or other stimuli [20–22]. Currently, a variety of organs-on-chips have been developed for drug screening [23, 24]. In particular, inspired by the physiological microstructures of hepatic sinusoid, several kinds of liver-on-a-chip have been constructed to simulate the liver microcirculation [25–27]. With these progresses, cell necrosis has been alleviated to a certain extent. However, most of these chips are just culturing cells on the surfaces of microchannels or still embedding cells in simple structural hydrogels, and the construction of an organ-on-a-chip with a hierarchical hydrogel to better mimic the real physiological microstructures is unexplored.

In this study, we employed the hierarchical hydrogel system for the liver cancer cell culture, organ-on-a-chip construction, and drug screening. Through a bottom-up self-assembly method, the hierarchical hydrogel was obtained by fully filling the gap between SiO_2_ nanoparticles and the customized microcolumn array template. The generated hierarchical hydrogel had ordered micro-nano structure, where the micropores were arranged in array and the ordered nanopores were distributed around each micropore. On the macroscopic level, the size of cell spheroids could be precisely controlled by micropores, while on the microscopic level, adequate nutrient supply of cell spheroids could be realized through nanopores. Benefiting from synergistic effect of these two levels, the size of cell spheroids cultured in the hierarchical hydrogel was more uniform and had a higher cell survival rate than those cultured in two-dimensional (2D) surfaces or simply microstructure hydrogels. More attractively, by integrating the hierarchical hydrogel into a multichannel concentration gradient microfluidic chip, a functional liver cancer-on-a-chip was constructed for high-throughput drug screening with good repeatability and high accuracy. These features make the hierarchical hydrogel system and its integrated cancer-on-a-chip an excellent platform for drug screening in personalized medicine.

## 2. Results and Discussion

**Figure 2:**
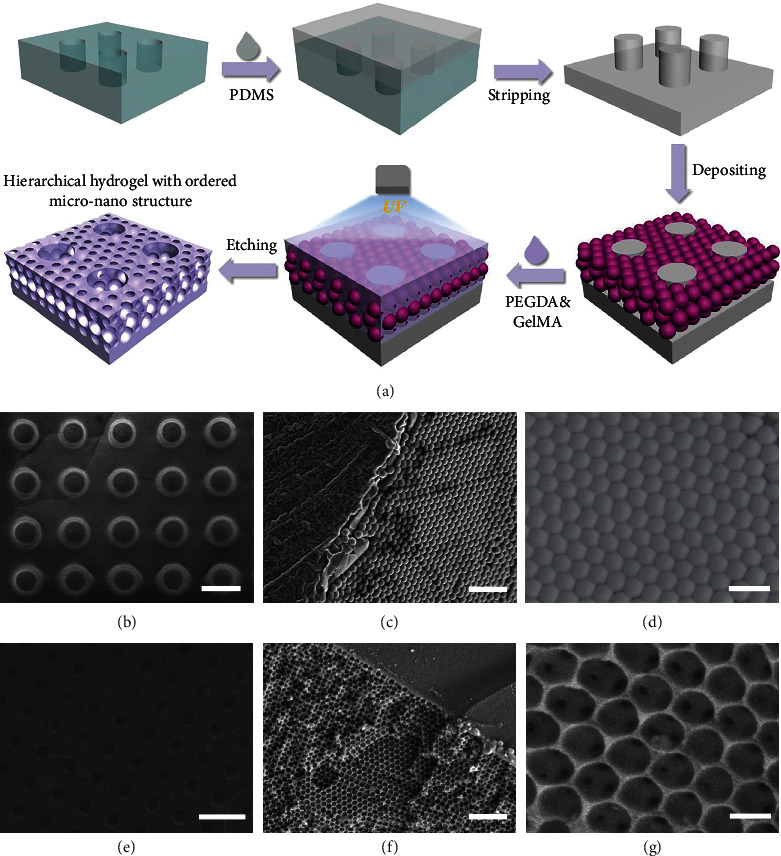
(a) Schematic of fabrication of the hierarchical hydrogel with ordered micro-nano structure. (b–d) SEM images of the macroscopic (b), microscopic side (c), and surface (d) of the nanoparticles deposited on PDMS microarray template. Scale bars = 400 *μ*m in (b), 3 *μ*m in (c), and 500 nm in (d). (e–g) SEM images of the macroscopic (e), microscopic side (f), and surface (g) of the hierarchical hydrogel with ordered micro-nano structure. Scale bars = 800 *μ*m in (e), 3 *μ*m in (f), and 400 nm in (g).

In a typical experiment, the hierarchical hydrogel with ordered micro-nano structure was fabricated by template replication method, as shown in Figure 2(a). In brief, first, a complete and smooth PDMS microarray was obtained through replicating the customized PMMA template (Figure S1). Then, ethanol solution containing silicon dioxide (SiO_2_) nanoparticles was dropped onto the PDMS microarray (Figure S2). After evaporation of the ethanol, self-assembled SiO_2_ nanoparticles were formed on the PDMS microarray. Next, a pregel containing polyethylene glycol diacrylate (PEGDA) and methacrylate gelatin (GelMA) hydrogels was completely filled into the gaps between nanoparticles and microarray. After the pregel was polymerized with ultraviolet (UV) light, the hydrogel was mechanically peeled off. Finally, the hierarchical hydrogel with ordered micro-nano structure was obtained by etching the nanoparticles with hydrofluoric acid (HF).

SiO_2_ nanoparticles can self-assemble under van der Waals forces, hydrogen bonds, hydrophobic interactions, or other noncovalent forces and form hexagonal close-packed structures on a two-dimensional (2D) substrate. In this research, PDMS microarray was used as the substrate for nanoparticle self-assembly instead of planar surfaces. In order to figure out the self-assembly of nanoparticles on the PDMS microarray, a scanning electron microscopy (SEM) was chosen to characterize the structure of nanoparticles (Figures 2(b)–2(d)). From the side and surface perspectives, the nanoparticles presented a closely packed face-centered cubic structure, with a multilayer structure on the surface of the PDMS microarray. Then, the hydrogel was filled into the gaps between the nanoparticles and microarray under capillary action, and the hierarchical hydrogel with ordered micro-nano structure was obtained after etching the nanoparticles. To avoid the collapse of nanopores, PEGDA with high mechanical strength was added to provide sufficient supporting force. SEM images showed that the micropores were arranged neatly, and there existed ordered nanopores among the micropores (Figures 2(e)–2(g)). Meanwhile, the hierarchical hydrogel showed unique photonic band gaps (PBGs) before and after corrosion of nanoparticles due to the ordered nanostructure. This resulted in structure color and characteristic reflection peaks (Figure S3), which followed Bragg's equation:

(1) $$\begin{array}{c} \lambda =1.633{dn}_{\text{average}}, \end{array}$$

where *λ* is the characteristic reflection peak, *d* is the center-to-center distance between neighboring nanoparticles, and *n*_average_ indicates the average refractive index of the hydrogels.

Generally, a lower concentration of GelMA hydrogel has a larger pore size and higher porosity, which have a positive effect on the proliferation, differentiation, migration, and adhesion of cells attached or encapsulated in it [28–30]. However, with the decrease of GelMA concentration, the mechanical property of hydrogel decreased, leading to nanopore collapse, making it difficult to prepare the hierarchical hydrogel with ordered micro-nano structure. Thus, PEGDA hydrogel was added to improve the mechanical strength of the entire hydrogel system [31, 32]. The final hydrogel system consisted of 10% PEGDA (*v*/*v*) and 10% GelMA (*w*/*v*). 3-(4,5-Dimethylthiazol-2-yl)-2,5-diphenyltetrazolium bromide (MTT) method was selected to assay the biocompatibility of prepared hydrogel (Figure S4). Fluorescence images showed that 3T3 cells had good morphological characteristics and maintained a high survival rate. Compared with the control group (2D plate), the cell viability of the hierarchical hydrogel group remained above 95% from day 1 to day 3, which indicated that the hierarchical hydrogel with ordered micro-nano structure had good biocompatibility and could be used for subsequent cell culture.

**Figure 3:**
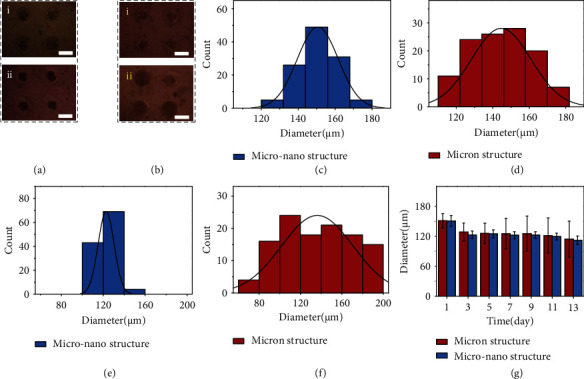
(a, b) HepG2 cells grown on the hierarchical hydrogel with ordered micro-nano structure (a) and the hydrogel with micron structure (b) for (i) 1 day and (ii) 7 days. (c, d) The size distribution of HepG2 cell spheroids grown on the hierarchical hydrogel with ordered micro-nano structure (c) and the hydrogel with micron structure (d) for 1 day. (e, f) The size distribution of HepG2 cell spheroids grown on the hierarchical hydrogel with ordered micro-nano structure (e) and the hydrogel with micron structure (f) for 7 days. (g) The average size of HepG2 cell spheroids grown on the hierarchical hydrogel with ordered micro-nano structure and the hydrogel with micron structure for 1, 3, 5, 7, 9, 11, and 13 days. Scale bars = 200 *μ*m in (a) and (b).

To explore the biological application of the hierarchical hydrogel, the hierarchical hydrogel with micro-nano structure was used to culture HepG2 cell spheroids. Due to the ordered nanopores, oxygen and nutrients can circulate more evenly through the micropores and be absorbed by cells, and the metabolites produced by cells can be completely transported out. In order to verify and highlight the advantages of the hierarchical hydrogel with micro-nano structure in 3D cell culture, HepG2 cells were cultured in a hydrogel with only micron structure, as a control group for comparative analysis. The formation of HepG2 cell spheroids was recorded by an optical microscope at day 1, 3, 5, 7, 9, 11, and 13 of culture (Figures S5 and S6). The ImageJ software was applied to measure and analyze the size of cell spheroids (Figure 3). To show the advantages of micro-nano structure in terms of size uniformity in long-term culture more intuitively, the size distribution of HepG2 cell spheroids on day 1 and day 7 was chosen for comparison (Figures 3(a)–3(f)). On the first day, the size of the spheroids grown in the two structures was about 150 *μ*m. In the hydrogel with micro-nano structure, the size of the spheroids on day 7 concentrated at 120 *μ*m, while in the hydrogel with microstructure, the distribution of size on day 7 was between 60 and 200 *μ*m. These results might be caused by the nanopores, which supplied sufficient oxygen and nutrients. In addition, with the increase of culture time, the size of spheroids in the hydrogel with micro-nano structure gradually decreased as the connections between cells became closer, which was similar to the previous report [33, 34]. The uniformity of the cell spheroids is critical to the repeatability and accuracy of drug screening [35], so it can be foreseen that the hierarchical hydrogel with micro-nano structure will have a wider application prospect.

**Figure 4:**
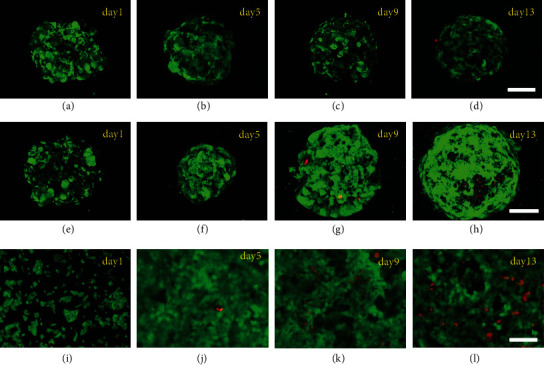
Live/dead staining of cells at days 1, 5, 9, and 13. (a–d) Confocal images of cells growing on the hierarchical hydrogel with ordered micro-nano structure. Scale bar = 60 *μ*m. (e–h) Confocal images of cells growing on the hydrogel with micron structure. Scale bar = 45 *μ*m. (i–l) Fluorescence images of cells growing on the 2D plane. Scale bar = 50 *μ*m.

In addition to controlling the uniformity of cell spheroids, the hierarchical hydrogel with micro-nano structure also had a positive effect on the long-term cell culture. To confirm this positive impact, the hydrogel with micron structure and 2D culture were selected as the control groups (Figure 4). It has been demonstrated that during long-term culture of spheroids, the cells in the middle often die due to the lack of oxygen and nutrients. To observe the survival status of cells inside the cell spheroids, the cells were stained with calcein-AM and propidium iodide (PI) and recorded under a confocal microscope and a fluorescence microscope. It was found that in the hydrogel with micro-nano structure, cell apoptosis did not happen until the 13th day at the edge of the cell spheroids (Figures 4(a)–4(d)), while in the hydrogel with micron structure, apoptosis occurred on the 9th day, and by the 13th day, a large number of apoptotic cells appeared from the lower hemisphere to the middle (Figures 4(e)–4(h)). Besides, in 2D culture, apoptosis appeared on the 5th day, and the rate of apoptosis increased with the prolongation of culture time (Figures 4(i)–4(l)). These results indicated that the hierarchical hydrogel with micro-nano structure significantly improved the occurrence of cell necrosis, which depended on the orderly arranged nanopores around the micropores so that the spheroids could obtain sufficient oxygen and nutrients. In addition, the nanopores also reduced the distance that oxygen and nutrients reached intermediate cells, greatly improving the survival rate of cells in long-term culture. In contrast, in the hydrogel with micron structure, the transport of nutrients was mainly dependent on the cells in the upper hemisphere, so the cells in the lower hemisphere were prone to necrosis in long-term culture. On the other hand, cells in 2D culture only attach on the side that contacted with the culture dish, which can not simulate the growth mode in vivo and affect cell proliferation and apoptosis over time. The MTT results (Figure S7) showed that the cell viability of these three groups was all increasing during the continuous culture process. Among them, the cell viability of the hydrogel with micro-nano structure was higher than that of the other two groups, while the cell viability of the hydrogel with micron structure was higher than 2D culture. These results were consistent with images of live/dead staining (Figure 4), highlighting the positive impact of layered hydrogels with micro-nano structures on long-term cell culture.

**Figure 5:**
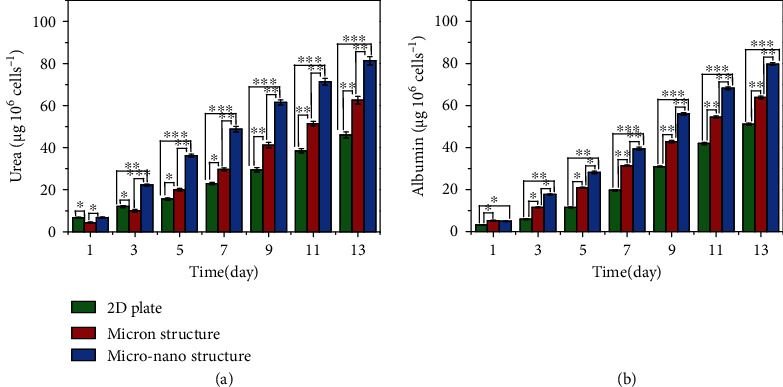
The metabolic functions of HepG2 cells cultured on a 2D plate, the hydrogel with micron structure, and the hierarchical hydrogel with ordered micro-nano structure: (a) urea synthesis and (b) albumin secretion. ^∗^*p* < 0.05, ^∗∗^*p* < 0.01, and ^∗∗∗^*p* < 0.001.

To further prove the advantages of our hierarchical hydrogel with ordered micro-nano structure in long-term cell culture, the metabolic functions of HepG2 cells were analyzed under the above three culture conditions. The urea synthesis and albumin secretion of HepG2 cells were quantitatively measured (Figure 5). In the continuous culture process, the synthesis of urea and secretion of albumin were significantly different among groups (*p* < 0.05). The urea synthesis and albumin secretion of cells cultured in hydrogel with micro-nano structure were greater than those of 2D and hydrogel with micron structure. This was because compared with the other two groups, the micro-nano structure of the hierarchical hydrogel more truly mimicked the cell growth microenvironment in vivo, thus promoting cell function and activity [36]. These data further demonstrated the advantages of the hierarchical hydrogel with micro-nano structure in cell culture.

**Figure 6:**
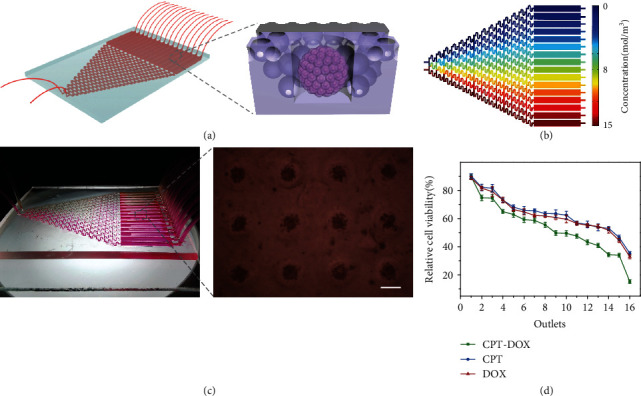
The applications of the hierarchical hydrogel with ordered micro-nano structure in a liver cancer-on-a-chip system. (a) Schematic of the construction of the liver cancer-on-a-chip. (b) Concentration gradient simulation of the liver cancer-on-a-chip. (c) Side image of the liver cancer-on-a-chip. Scale bar = 200 *μ*m. (d) Drug screening results of DOX, CPT, and DOX-CPT.

To achieve high-throughput drug screening, organ-on-a-chip technology was combined with hierarchical hydrogel to construct a liver cancer-on-a-chip (Figure 6(a)). As a bionic technology, the organ-on-a-chip can accurately control biochemical factors, mechanical stress, and other parameters, thereby simulating the smallest functional unit of human organs in vitro [23, 37]. In addition, the organ-on-a-chip also has the characteristics of low loss, high throughput, and integration, so it is regarded as a bionic, energy-saving, and efficient platform that can be used for physiology research and drug development [24, 26]. The designed liver cancer-on-a-chip included a concentration gradient module and a drug response module. The classic “Christmas tree” model was selected to construct the concentration gradient module, which was designed to generate 16 drug concentrations. The drug response module was composed of multiple independent rectangular channels, allowing cells to interact with different concentrations of drugs without interfering with each other (Figure S8).

To find the appropriate inlet velocity more simply and quickly, numerical simulation was carried out using COMSOL Multiphysics (Figure 6(b)). Based on the Navier-Stokes equation,

(2) $$\begin{array}{c} \rho \left(\frac{\partial \upsilon }{\partial t}+\upsilon \cdot \nabla \upsilon \right)=-\nabla p+\nabla \cdot \left(\mu \left(\nabla \upsilon +{\left(\nabla \upsilon \right)}^{\text{T}}\right)-\frac{2}{3}\mu \left(\nabla \cdot \upsilon \right)I\right)+F, \end{array}$$

where *ρ* is the density, *υ* is the velocity vector, *p* is the pressure, *μ* is the power viscosity of the fluid, *T* is the absolute temperature, and *F* is the volume force vector; different simulation results were obtained by setting different inlet velocities (Figure S9). When the speed was 10^−4^ m/s, the fluids flowed into the next-stage outlet without complete mixing, while when the speed was 10^−5^ m/s, the fluids could be fully mixed and entered the next-stage outlet. After optimization, the concentration gradient matching with the theoretical value was obtained. According to the optimized speed, the medium (pink) and the drug (yellow) were pumped into two inlets (Figure 6(c)), and the color of the 16 outlet solutions changed obviously in a gradient, which was consistent with the theoretical value and the simulated value. The antitumor drugs such as doxorubicin hydrochloride (DOX) and camptothecin (CPT) were injected for drug screening (Figure 6(d)). The quantitative results indicated that with the increase of DOX or CPT concentration, the cell viability continued to decrease. In addition, with the addition of CPT, the cell viability of the CPT-DOX group was lower than that of the DOX group and the CPT group under the synergistic action of drugs, which was consistent with related reports [38, 39]. The above results proved the effectiveness of the liver cancer-on-a-chip constructed by integrating the hierarchical hydrogel with ordered micro-nano structure and microfluidic chip in drug screening.

## 3. Conclusion

In conclusion, we have proposed a novel hierarchical hydrogel system with ordered micro-nano structure for the liver cancer cell culture, organ-on-a-chip construction, and drug screening. In such a hierarchical hydrogel, the micropores arranged in array could precisely control the size of cell spheroids, and the orderly distribution of nanopores around each micropore provided adequate nutrients for cells. Benefiting from the synergistic effect of these two structures, the generated cell spheroids could maintain a high survival rate in long-term culture. Besides, the high-throughput drug screening with good repeatability and high accuracy was achieved through a functional liver cancer-on-a-chip, which was prepared by combining the hierarchical hydrogel with a multichannel concentration gradient microfluidic chip. Thus, the designed hierarchical hydrogel with ordered micro-nano structure is expected to be an ideal platform for drug screening and widely used in the personalized medicine.

## 4. Materials and Methods

### 4.1. Materials

SiO_2_ nanoparticles were self-prepared through the Stöber method, and methacrylate gelatin (GelMA) was self-synthesized from gelatin [40]. Polyethylene glycol diacrylate (PEGDA), polydimethylsiloxane (PDMS), 2-hydroxy-2-methylpropiophenone (HMPP), dimethyl sulfoxide (DMSO), doxorubicin hydrochloride (DOX), and camptothecin (CPT) were bought from Sigma, USA. Hydrofluoric acid (HF) was gotten from Aladdin, Shanghai. HepG2 cells were obtained from the Institute of Biochemistry and Cell Biology, the Chinese Academy of Sciences, Shanghai, China. Calcein-AM and propidium iodide (PI) were purchased from Molecular Probes, USA. Phosphate balanced solution (PBS) (pH 7.4) was obtained from Gibco, USA. Albumin and urea assay kit were obtained from Nanjing Jiancheng Bioengineering Institute, China. Water used in all processes was purified through a Milli-Q system (Millipore) with a resistivity higher than 18 M*Ω* cm.

### 4.2. Construction of Hierarchical Hydrogel with Ordered Micro-Nano Structure

PDMS and curing agent were stirred at a weight ratio of 10 to 1 and put in a vacuum pump for defoaming. Then, the solution was poured onto a microarray template (200 *μ*m in diameter and 100 *μ*m in depth) and put into the vacuum pump to make the holes of the microarray template fully filled with the solution. Subsequently, the hybrid was placed in a 70°C oven for curing and finally mechanically stripped to obtain a polymer template. An ethanol solution containing SiO_2_ nanoparticles (35% *w*/*v*) was coated on the polymer template surface. After the ethanol evaporated, the prehydrogel solution with 10% PEGDA (*v*/*v*), 10% GelMA (*w*/*v*), and 1% HMPP (*v*/*v*) was poured onto the polymer template and filled the voids between template and SiO_2_ nanoparticles. Next, the prehydrogel solution was cured by UV light; the polymer template and the SiO_2_ nanoparticles were corroded with HF and rinsed for 3 times to obtain the hierarchical hydrogel with ordered micro-nano structure.

### 4.3. Cell Culture

The hierarchical hydrogel with micro-nano structure and the hydrogel with only micron structure were first disinfected under UV light for 6 h and soaked with PBS before culturing cells. 35 *μ*L of the HepG2 suspension with a concentration of 4 × 10^5^ cells/mL was dropped on the hydrogels to generate HepG2 cell spheroids, and cell suspension with the same volume and concentration was dropped on the surface of the 2D culture dish, and then, 2 mL of media was gently added. In order to determine the size of HepG2 cell spheroids, cell spheroids cultured in different structure hydrogels were recorded by a microscope on 1, 3, 5, 7, 9, 11, and 13 days, and the sphere diameter was calculated by ImageJ. After 1, 5, 9, and 13 days of culture, the cell spheroids were stained with calcein-AM (1 *μ*L/mL) and PI (2 *μ*L/mL) at 37°C for 20 min and imaged by an inverted fluorescence microscope and a confocal microscope. The secretion of albumin and synthesis of urea were measured by the albumin and urea assay kit.

### 4.4. Construction of Liver Cancer-on-a-Chip

The HepG2 cell spheroids cultured for 5 days in the hierarchical hydrogel with micro-nano structure were integrated into the designed chip. One inlet of the chip flowed into the culture medium through a microfluidic pump, and the other inlet flowed into different drugs through another microfluidic pump. Three groups of drug experiments were designed: DOX (6 mM) alone, CPT (1 mM) alone, and DOX combined with CPT. The drug concentrations of 1-16 outlets are as follows: DOX: 0 mM, 0.4 mM, 0.8 mM, 1.2 mM, 1.6 mM, 2 mM, 2.4 mM, 2.8 mM, 3.2 mM, 3.6 mM, 4 mM, 4.4 mM, 4.8 mM, 5.2 mM, 5.6 mM, and 6 mM; CPT: 0 mM, 0.07 mM, 0.13 mM, 0.2 mM, 0.27 mM, 0.33 mM, 0.4 mM, 0.47 mM, 0.53 mM, 0.6 mM, 0.67 mM, 0.73 mM, 0.8 mM, 0.87 mM, 0.93 mM, and 1 mM. After the drug concentration of the 16 outlets stabilized, the chip was placed in the incubator for 1 day. The medium containing 10% MTT solution was then injected through two inlets of the chip and incubated at 37°C for 4 h. Finally, DMSO was injected through the inlets to dissolve the crystals and then collected at 16 outlets, and the absorbance was measured through a microplate reader (Synergy HT, BioTek, USA).

### 4.5. Characterization

SEM images of microstructures of the self-assembled SiO_2_ nanoparticles and hierarchical hydrogel with ordered micro-nano structure were photoed by a scanning electron microscope (Hitachi S-3000N). The fluorescent images of cells cultured in 2D plate were captured by a fluorescence microscope (Olympus SZX16). Confocal microscopy images of cell spheroids were measured by a laser scanning microscope (Zeiss LSM700, Heidenheim, Germany). Microscopy images of HepG2 cell spheroids cultured in different structure hydrogels were recorded using an optical microscope (Olympus BX51) equipped with a color CCD camera (Olympus, DP30BW). Different simulation results at different inlet speeds were calculated by COMSOL Multiphysics 5.5.

## Data Availability

All data needed to evaluate the conclusions in the paper are present in the paper and/or the Supplementary Materials. Additional data related to this paper may be requested from the authors.

## Supplementary Materials

Supplementary Materials Figure S1: the optical images and SEM images of the PDMS temple. Figure S2: deposition of different volumes of SiO_2_ on PDMS template. Figure S3: the optical images and their characteristic reflection peaks before and after the corrosion of the hydrogel with different particle diameters of SiO_2_. Figure S4: fluorescence images of 3 T3 cells live staining on different days and MTT results from day 1 to day 3 of the hierarchical hydrogel with ordered micro-nano structure and the control group. Figure S5: schematic diagram of the hierarchical hydrogel with ordered micro-nano structure and the size of HepG2 cell spheroids on the hierarchical hydrogel with ordered micro-nano structure for different days. Figure S6: schematic diagram of the hydrogel with micron structure and the size of HepG2 cell spheroids on the hydrogel with micron structure for different days. Figure S7: MTT results of cells growing on the hierarchical hydrogel with ordered micro-nano structure, the hydrogel with micron structure, and the 2D plane for different days. Figure S8: the chip template design and optical images of the PDMS chip. Figure S9: simulation results at different speeds.
