# Supplementary material for: Hierarchical Hydrogels with Ordered Micro-Nano Structures for Cancer-on-a-Chip Construction
Source: Research (Wash D C). 2021 Dec 26;2021:9845679. doi: 10.34133/2021/9845679 (PMC8724685; doi:10.34133/2021/9845679)
Supplement: Supplementary Materials — Figure S1: the optical images and SEM images of the PDMS temple. Figure S2: deposition of different volumes of SiO2 on PDMS template. Figure S3: the optical images and their characteristic reflection peaks before and after the corrosion of the hydrogel with different particle diameters of SiO2. Figure S4: fluorescence images of 3 T3 cells live staining on different days and MTT results from day 1 to day 3 of the hierarchical hydrogel with ordered micro-nano structure and the control group. Figure S5: schematic diagram of the hierarchical hydrogel with ordered micro-nano structure and the size of HepG2 cell spheroids on the hierarchical hydrogel with ordered micro-nano structure for different days. Figure S6: schematic diagram of the hydrogel with micron structure and the size of HepG2 cell spheroids on the hydrogel with micron structure for different days. Figure S7: MTT results of cells growing on the hierarchical hydrogel with ordered micro-nano structure, the hydrogel with micron structure, and the 2D plane for different days. Figure S8: the chip template design and optical images of the PDMS chip. Figure S9: simulation results at different speeds. [file 9845679.f1.zip › Zhu_SI clean vision.docx]

Supplementary Materials for

**Hierarchical Hydrogel with Ordered Micro-nano Structure for Cancer-on-a-chip Construction**

Luyao Zhu, Changmin Shao, Hanxu Chen, Zhuoyue Chen, Yuanjin Zhao*

Figure S1. The optical images and SEM images of the PDMS temple.

Figure S2. Deposition of different volumes of SiO_2_ on PDMS template.

Figure S3. The optical images and their characteristic reflection peaks before and after the corrosion of the hydrogel with different particle diameters of SiO_2_.

Figure S4. Fluorescence images of 3T3 cells live staining on different days and MTT results from day1 to day3 of the hierarchical hydrogel with ordered micro-nano structure and the control group.

Figure S5. Schematic diagram of the hierarchical hydrogel with ordered micro-nano structure and the size of HepG2 cell spheroids on the hierarchical hydrogel with ordered micro-nano structure for different days.

Figure S6. Schematic diagram of the hydrogel with micron structure and the size of HepG2 cell spheroids on the hydrogel with micron structure for different days.

Figure S7. MTT results of cells growing on the hierarchical hydrogel with ordered micro-nano structure, the hydrogel with micron structure, and the 2D plane for different days.

Figure S8. The chip template design and optical images of the PDMS chip.

Figure S9. Simulation results at different speeds.


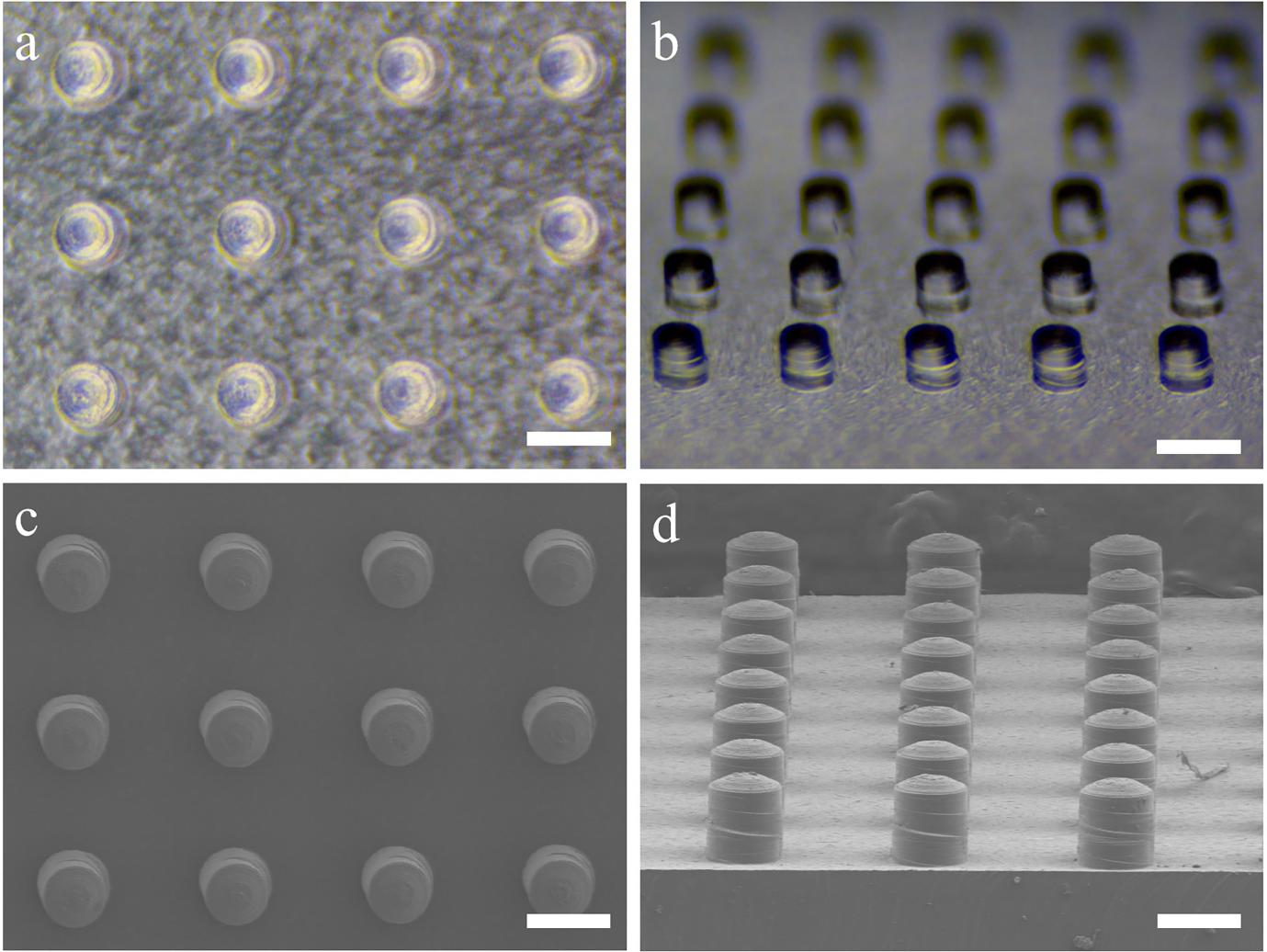


**Figure S1.** (a-b) Optical images of the front (a) and side (b) of the PDMS temple. Scale bar = 150 µm. (c-d) SEM images of the front (c) and side (d) of the PDMS temple. Scale bar = 120 µm.


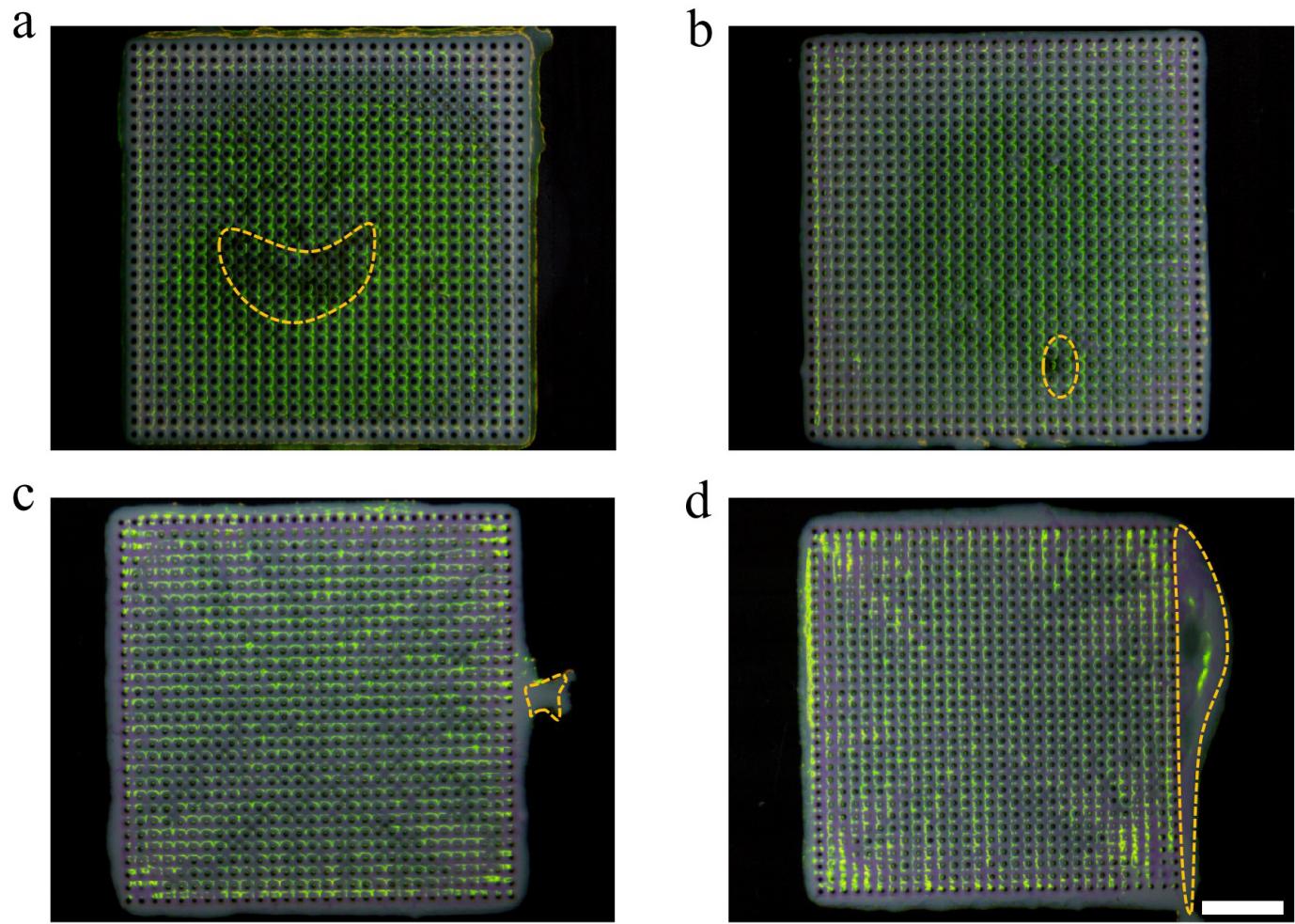


**Figure S2.** Deposition of different volumes of SiO_2_ on PDMS template. (a) 20 µL (b) 30 µL

(c) 40 µL (d) 50 µL. The yellow dotted line in (a) and (b) indicates that the SiO_2_ nanoparticles are not completely filled, in (c) and (d) indicates that the SiO_2_ nanoparticles are overfilled. Scale bar = 2.6 mm.


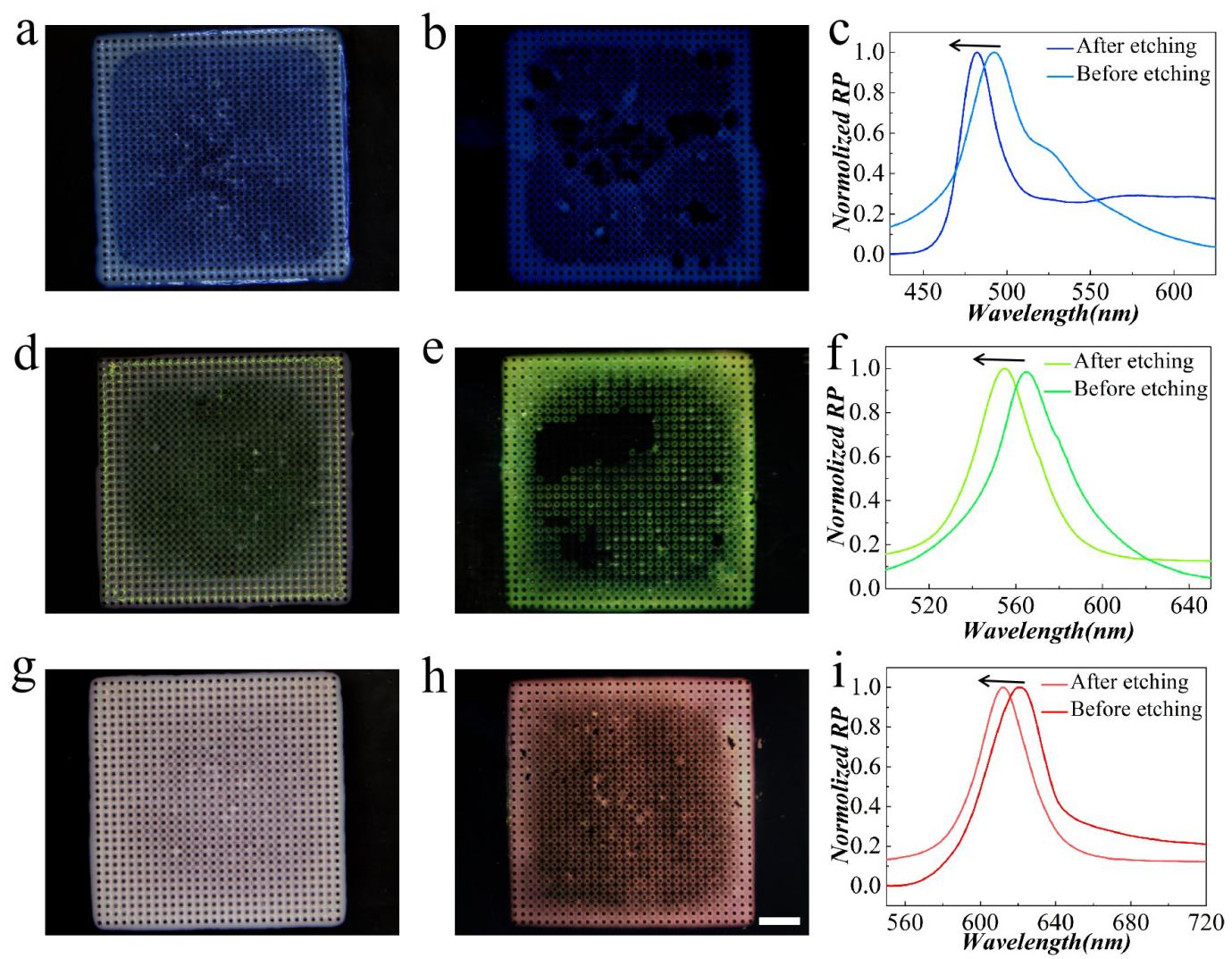


**Figure S3.** The optical images and their characteristic reflection peaks before and after the corrosion of the hydrogel with different particle diameters of SiO_2_. (a-c) particle diameter of SiO_2_ is 490 nm. (d-f) particle diameter of SiO_2_ is 570 nm. (g-i) particle diameter of SiO_2_ is 630 nm. Scale bar = 2 mm.


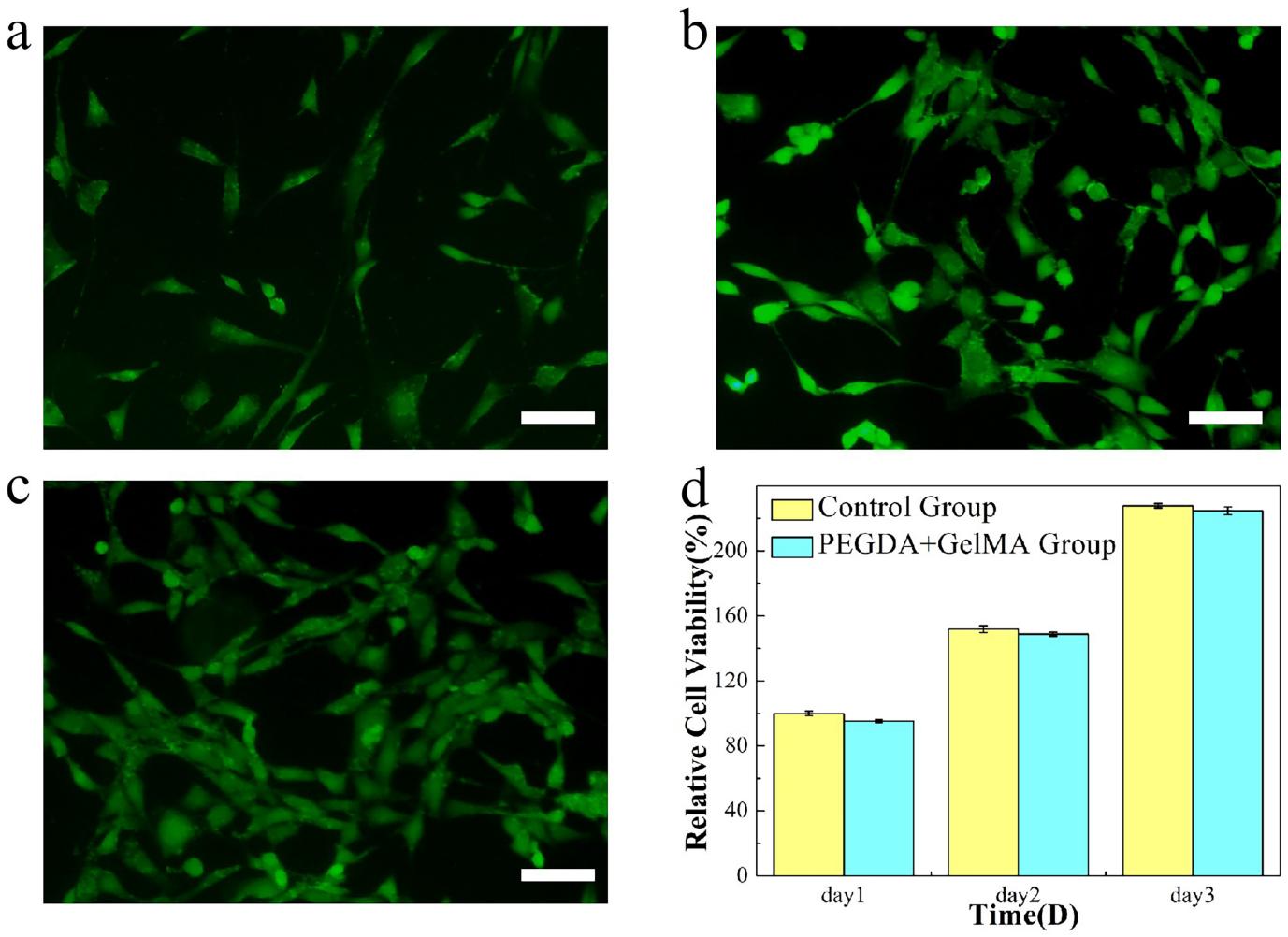


**Figure S4.** Fluorescence images of 3T3 cells live staining on day1 (a), day2 (b) and day3 (c). Scale bar = 100 µm. (d) MTT results from day1 to day3 of the hierarchical hydrogel with ordered micro-nano structure and the control group (well plates).


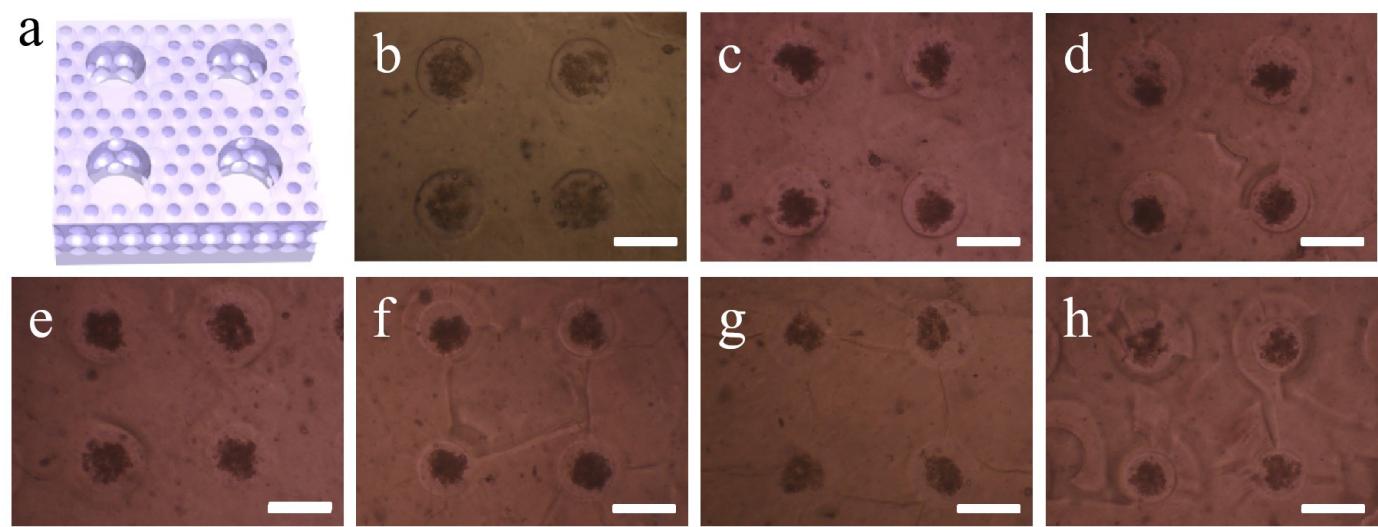


**Figure S5.** (a) Schematic diagram of the hierarchical hydrogel with ordered micro-nano structure. (b-h) The size of HepG2 cell spheroids on the hierarchical hydrogel with ordered micro-nano structure for (b) 1 day (c) 3 days (d) 5 days (e) 7 days (f) 9 days (g) 11 days (h) 13 days. Scale bar = 150 µm.

**
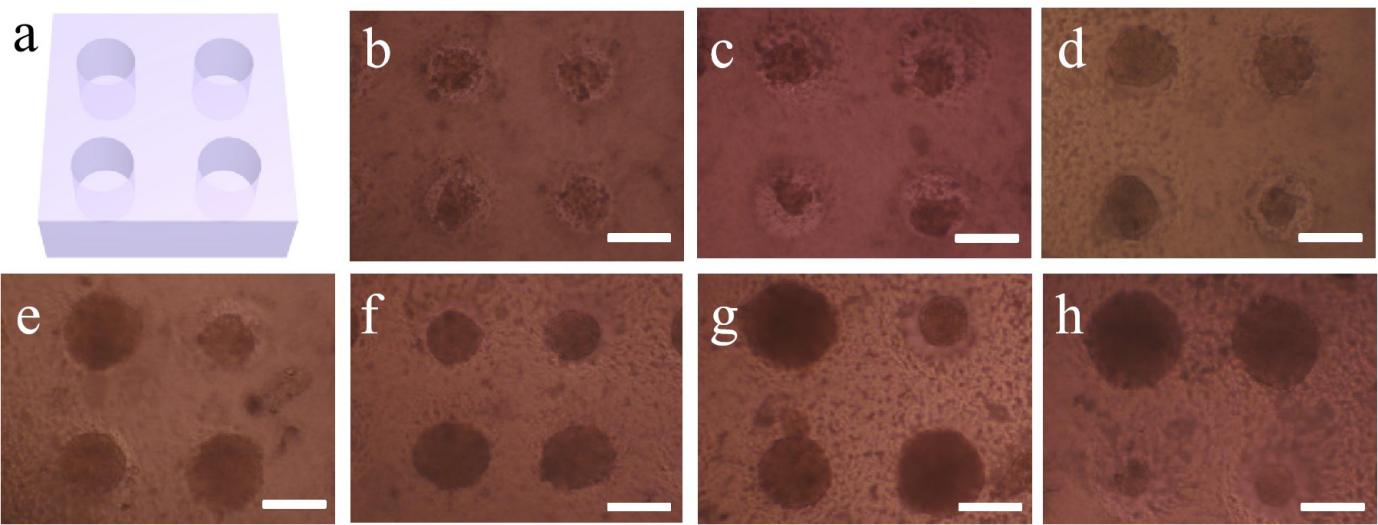
**

**Figure S6.** (a) Schematic diagram of the hydrogel with micron structure. (b-h) The size of HepG2 cell spheroids on the hydrogel with micron structure for (b) 1 day (c) 3 days (d) 5 days (e) 7 days (f) 9 days (g) 11 days (h) 13 days. Scale bar = 150 µm.


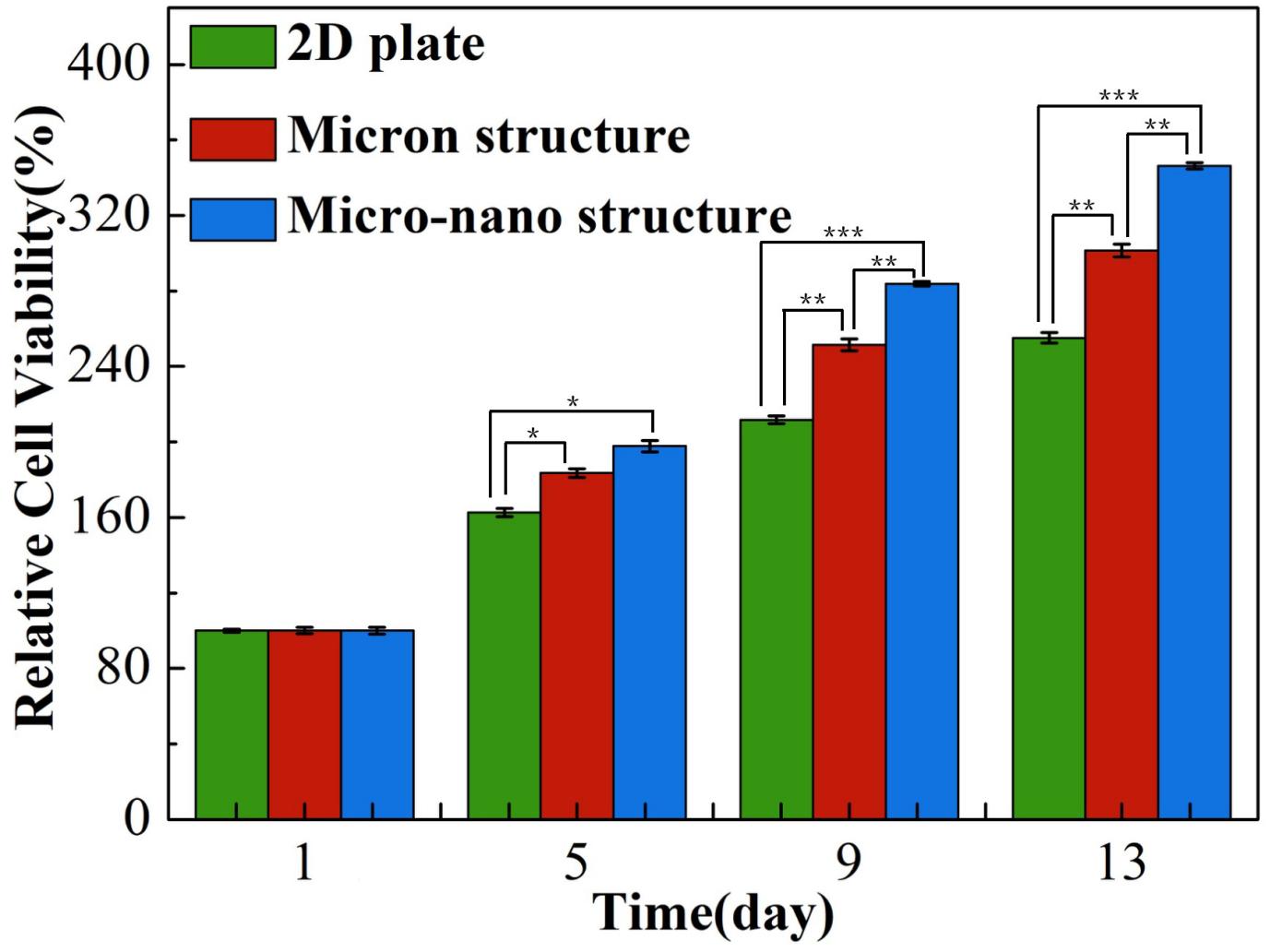


**Figure S7.** MTT results of cells growing on the hierarchical hydrogel with ordered micro-nano structure, the hydrogel with micron structure, and the 2D plane for 1, 5, 9, and 13 days. *p < 0.05, **p < 0.01, ***p < 0.001.


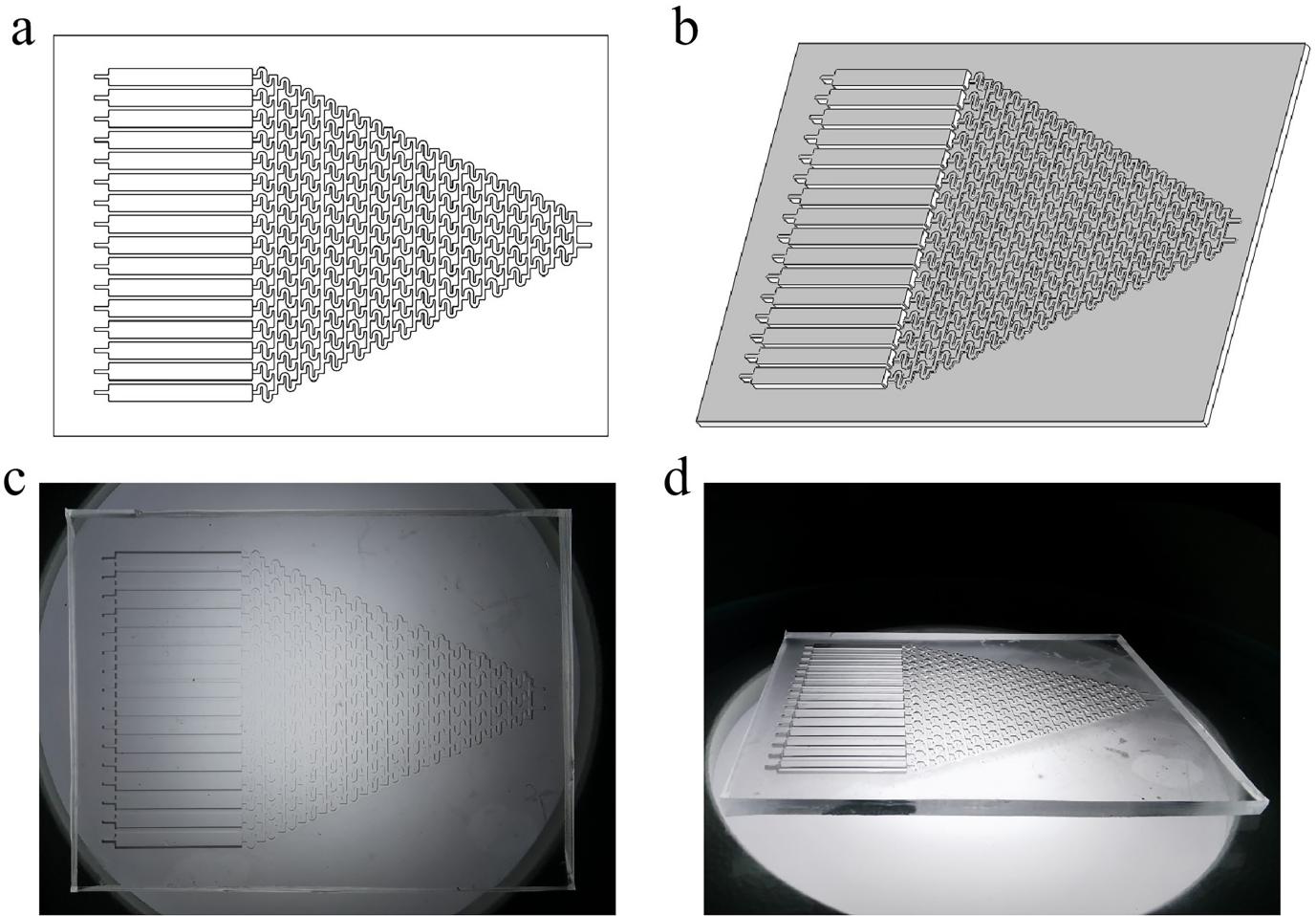


**Figure S8.** The (a) front and (b) side of the chip template design. The (c) front and (d) side of the PDMS chip.


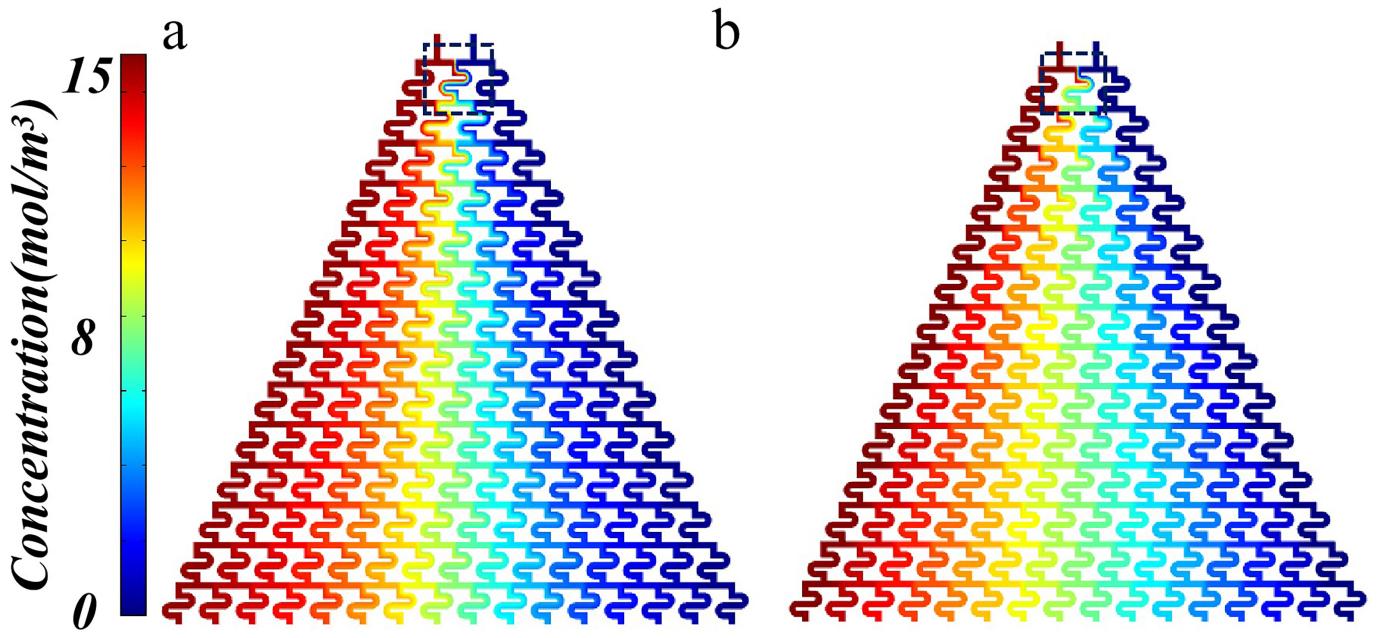


**Figure S9.** Simulation results at different speeds. (a)10^-4^ m/s (b)10^-5^ m/s.
